# Case Report: Facial nerve schwannoma: comprehensive surgical management with nerve reconstruction and hearing rehabilitation

**DOI:** 10.3389/fsurg.2025.1734804

**Published:** 2026-01-13

**Authors:** F. Deffner, A. Aschendorff, S. Arndt, S. U. Eisenhardt, F. Hassepaß, M. C. Ketterer

**Affiliations:** Department of Oto-Rhino-Laryngology, Medical Center – University of Freiburg, Freiburg, Germany

**Keywords:** facial nerve paresis, facial nerve schwannoma, nerve reconstruction, otologic surgery, surgical management

## Abstract

Facial nerve schwannomas are rare tumors that pose diagnostic and surgical challenges. We report a 17-year-old female with progressive right-sided facial paresis initially misdiagnosed as Bell's palsy. MRI revealed a contrast-enhancing lesion of the facial nerve. She underwent a two-stage surgery: tumor resection via mastoidectomy and hearing rehabilitation, followed by facial nerve reconstruction using masseteric-to-facial nerve transfer and cross-face sural grafting. At nine months postoperatively, facial function improved from House-Brackmann grade V to III, and hearing was preserved. Early imaging and multidisciplinary management can enable complete tumor resection with functional restoration and favorable outcomes.

## Case history

A 17-year-old female presented with progressive right-sided facial nerve paresis that had first appeared four months prior. An initial diagnosis of Bell's palsy was made, and oral prednisolone therapy was administered without clinical improvement. Serologic testing for multiple pathogens, including Borrelia burgdorferi, was negative. The patient did not report hearing loss or vertigo.

## Clinical findings

**Figure 1:**
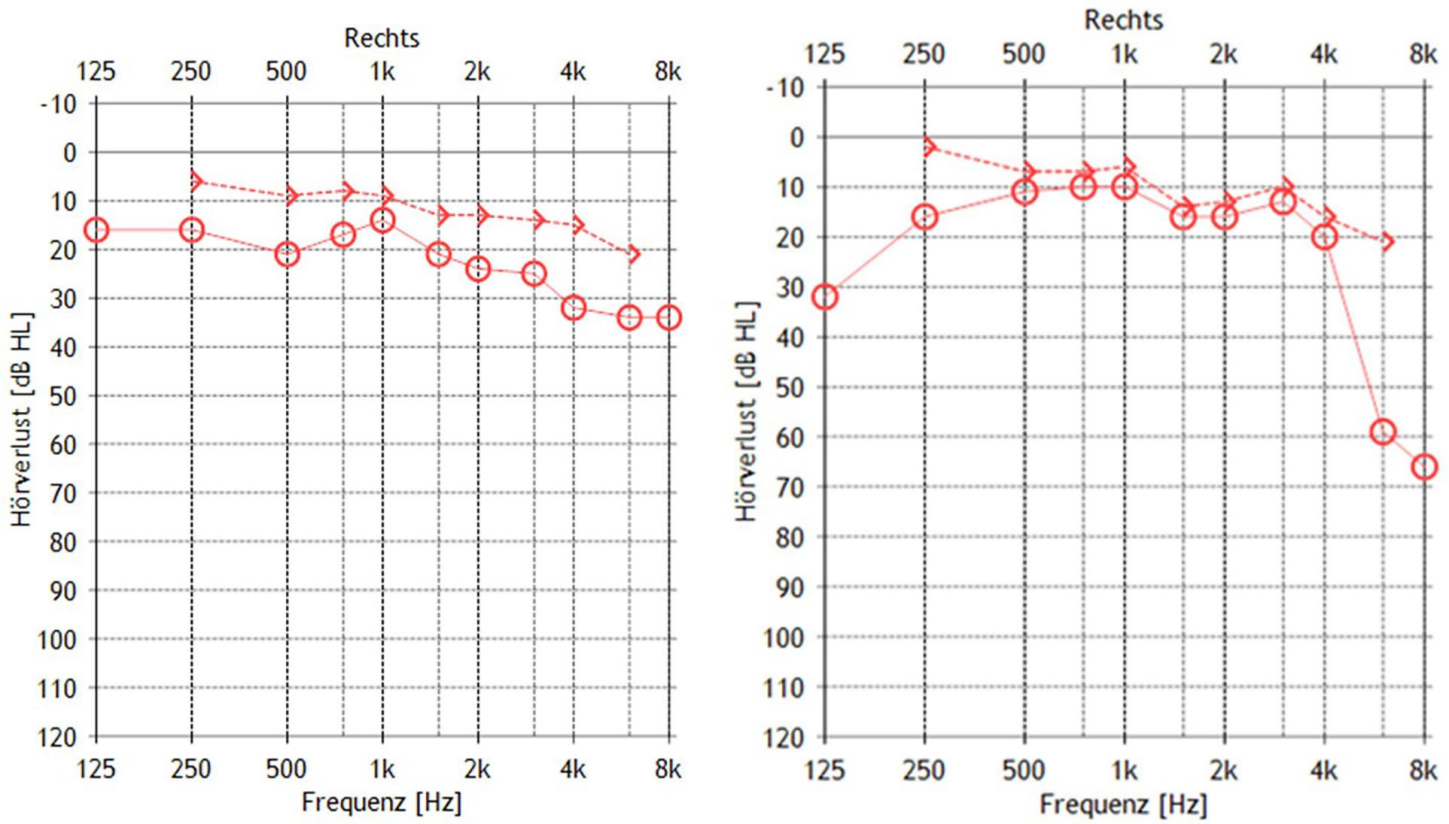
BC and AC Pure-tone tresholds of the right ear. Left: Preoperative results. Right: Postoperative results 9 months after reconstruction.

Examination revealed a House-Brackmann grade V facial nerve paresis. Otoscopic inspection showed a bulging tympanic membrane in the posterosuperior quadrant with a negative Valsalva maneuver. Pure-tone audiometry demonstrated an air–bone gap of 10 dB across the entire frequency range (Figure 1, left), without impairment of speech discrimination. Tympanometry revealed a flattened curve. Video-based head impulse testing showed normal vestibulo-ocular reflex gains in all three semicircular canals.

Cranial Magnet Resonance Imaging (cMRI) demonstrated a thickened and contrast-enhancing epitympanic segment of the facial nerve extending from the geniculate ganglion on the right side.

**Figure 2:**
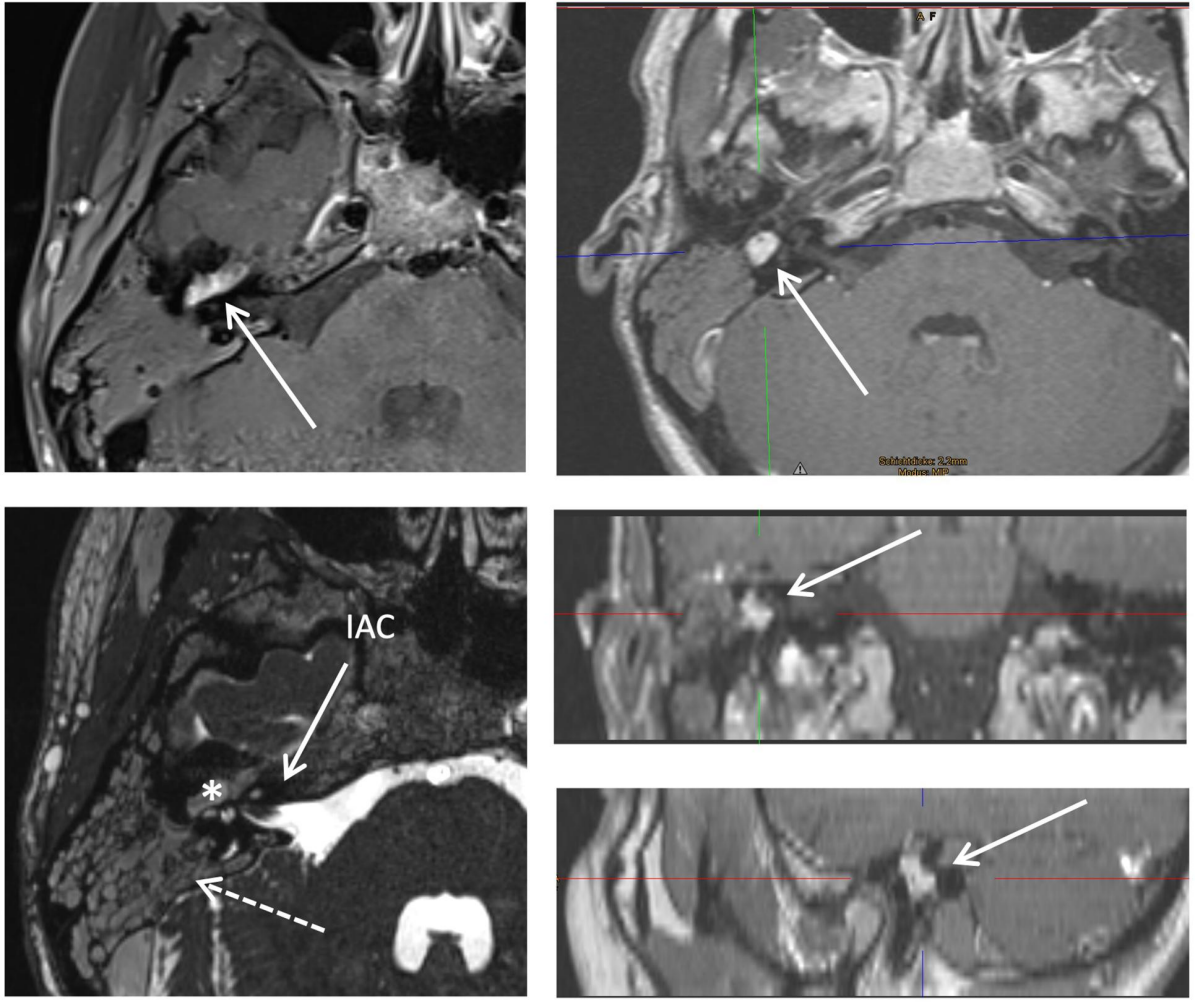
Preoperative cMRI. Top left: Axial T1-weighted sequence showing thickening of the mastoid and contrast enhancement of the epitympanic segment of the facial nerve (arrow). Bottom left: Axial high-resolution T2-weighted 3D sequence demonstrating complete opacification of the right mastoid (dashed arrow) and a ventilation disorder caused by a sharply circumscribed hyperintense mass (*) in the mastoid antrum. IAC, internal auditory canal. Top, middle and bottom right: Axial, coronal and sagittal T1-weighted sequence showing schwannoma of the facial nerve (arrows).

**Figure 3:**
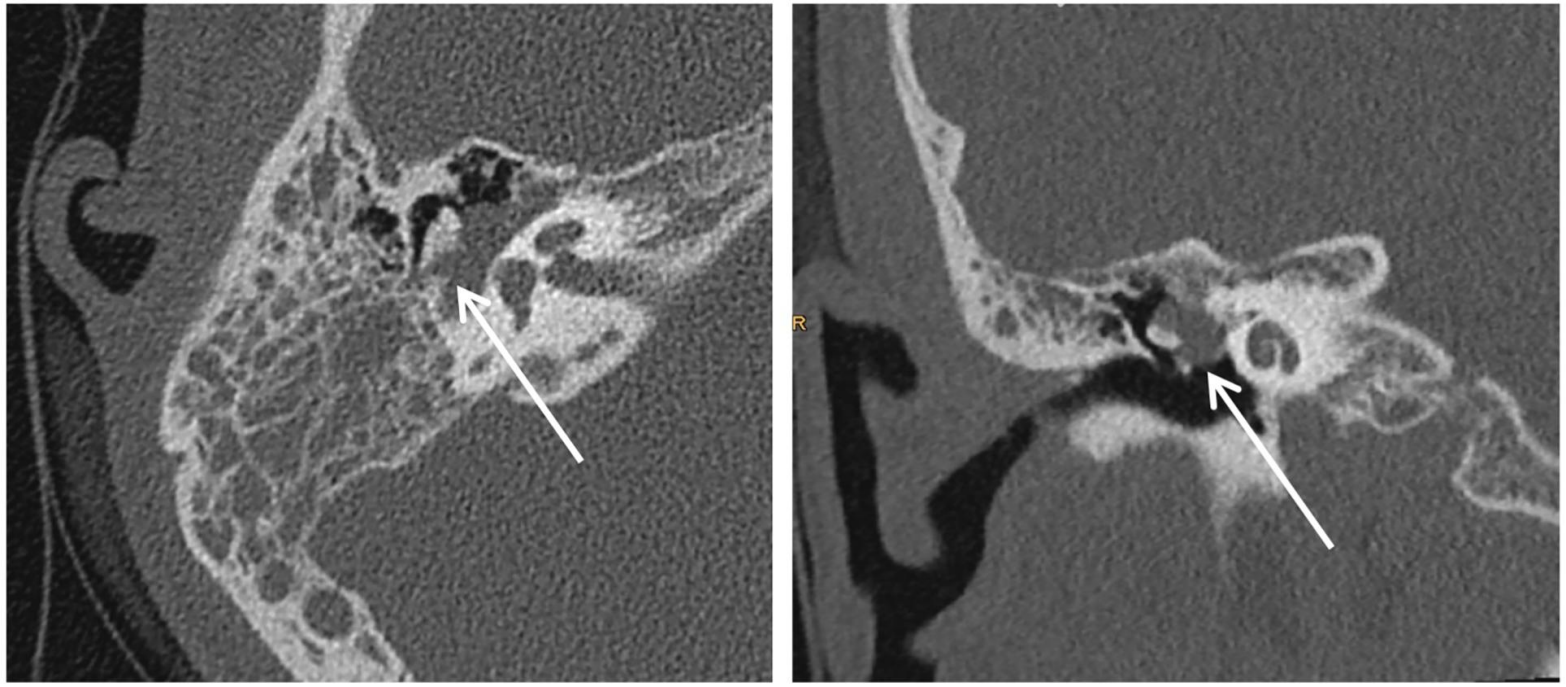
CT scan of the right temporal bone (right axial and left coronal): soft tissue opacity of the right mesotympanum and hypotympanum (arrows). No signs of erosion of the ossicles. Complete opacification of the air cells of the right mastoid.

High-resolution 3D T2-weighted imaging showed complete opacification of the mastoid, interpreted as impaired ventilation due to a mass in the mastoid antrum (Figure 2). CT scan of the right temporal bone showed soft tissue opacity of the right mesotympanum and hypotympanum and complete opacification of the air cells of the right mastoid (Figure 3).

## Diagnosis

These findings were suggestive of a facial nerve schwannoma. At an interdisciplinary skull base board meeting, surgical resection of the tumor by an otologic surgeon was recommended, followed by facial nerve reconstruction by a plastic surgeon.

## Treatment and outcome

Given the tumor's extensive involvement, a two-stage surgical approach was selected in consultation with the patient. The first stage consisted of tumor resection via mastoidectomy with posterior tympanotomy. To improve surgical access, the malleus and incus were removed, allowing for complete tumor excision. A simultaneous hearing rehabilitation was performed using type III tympanoplasty with incus interposition, resulting in very satisfactory hearing thresholds on follow-up pure-tone audiometry at four weeks postoperatively (Figure 1, right).

**Figure 4:**
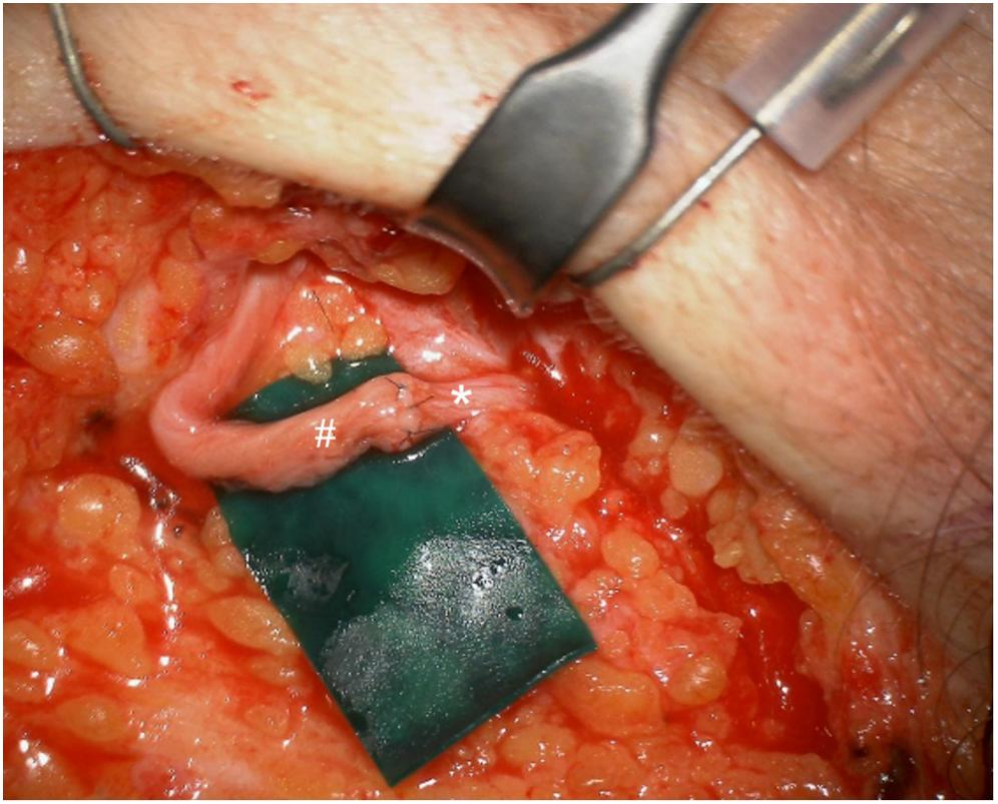
Cross-face nerve graft: connection of the sural nerve graft to the zygomatic branch of the facial nerve. # – sural nerve, * – zygomatic branch of the facial nerve.

In the second stage, performed six weeks later, facial nerve reconstruction was achieved by transferring the masseteric nerve (a motor branch of the mandibular division of the trigeminal nerve, V3) to the main trunk of the facial nerve. Additionally, a segment of the sural nerve was harvested from the lower leg and used as a cross-face nerve graft, anastomosed bilaterally to augment the function of the zygomatic branch (Figure 4).

At nine months postoperatively, the patient exhibited an improved facial function, now corresponding to House-Brackmann grade III. This improvement is primarily attributable to the masseteric nerve coaptation, while an effect of the cross-face nerve graft on resting tone and spontaneous smile development can be expected no earlier than one year postoperatively.

An MRI with contrast was performed 12 months after surgery and showed no evidence of recurrence.

## Discussion

Although facial nerve schwannomas are rare, they pose significant diagnostic and therapeutic challenges due to their anatomical complexity and the risk of functional deficits. In this case, the initial misdiagnosis of Bell's palsy underscores the difficulty in differentiating idiopathic from structural causes of facial nerve paresis based solely on early clinical presentation. The lack of response to corticosteroid therapy and negative infectious workup appropriately prompted advanced imaging, which revealed the true pathology (1).

MRI was critical for diagnosis, with characteristic findings of nerve thickening and contrast enhancement. High-resolution T2-weighted imaging further delineated a water signal in the mastoids, likely secondary to impaired aeration caused by the tumor. These findings are consistent with typical radiographic features of facial nerve schwannomas described in the literature (2, 3).

The extent and location of the tumor in our patient, together with the anticipated need for complex facial nerve reconstruction, led our interdisciplinary team to favor a staged approach. Involvement of the geniculate ganglion and the labyrinthine segment are key determinants in facial nerve schwannoma management, as they not only increase the likelihood of pronounced facial paresis but also significantly influence therapeutic decision-making (4, 5). An alternative management strategy would have been a one-stage approach with simultaneous tumor excision and immediate facial nerve grafting, which is more commonly applied in specialized centers. In most larger series, when the facial nerve requires sacrifice during schwannoma resection, reconstruction is performed during the same operation—typically using interposition cable grafting—to optimize functional outcomes and avoid the morbidity of multiple procedures (6–8).

The choice of mastoidectomy with posterior tympanotomy aligns with established surgical principles advocating for radical excision to reduce recurrence risk, while preserving neural integrity as much as possible (9, 10). Stereotactic radiosurgery is a relevant alternative for selected facial nerve schwannomas and offers good tumor control with generally preserved facial function. However, its role is limited in cases with advanced paresis, significant mass effect, or when immediate decompression and nerve reconstruction are required (11, 12).

Auditory function was preserved and successfully restored through concurrent type IIIa tympanoplasty using an incus interposition, demonstrating the viability of simultaneous hearing rehabilitation in selected cases (9). This highlights the importance of integrating auditory preservation into tumor resection strategies whenever feasible.

The second-stage nerve reconstruction employed both masseteric-to-facial nerve transfer and cross-face sural nerve grafting, representing advanced reconstructive options that aim to restore voluntary and spontaneous facial movements. Use of the masseteric nerve provides robust motor input, while cross-face grafting facilitates symmetry and mimetic function, a technique supported by increasing clinical evidence (10, 13). Compared with hypoglossal nerve transfer, the masseteric nerve offers several advantages for facial reanimation, including faster and stronger recovery of voluntary smile movement, minimal donor-site morbidity without tongue atrophy or speech and swallowing difficulties, and easier patient-controlled activation (14).

The patient's improvement to House-Brackmann grade III function at nine months postoperatively is encouraging and consistent with reported outcomes for facial nerve reconstruction following tumor resection, particularly when timely and technically precise interventions are employed (14, 15).

## Conclusion

This case highlights the importance of accurate early diagnosis, interdisciplinary treatment planning, and the application of modern reconstructive techniques in the management of facial nerve schwannomas. Comprehensive and timely intervention not only addresses the underlying pathology but also enables substantial functional recovery and improved quality of life in affected patients.

## Data Availability

The original contributions presented in the study are included in the article/Supplementary Material, further inquiries can be directed to the corresponding authors.
